# Preparation and Electrochemical Characteristics of the Co-Doped Li_7_La_3_Zr_2_O_12_ Solid Electrolyte with Fe^3+^ and Bi^3+^

**DOI:** 10.3390/molecules30092028

**Published:** 2025-05-02

**Authors:** Jialu Qu, Xingyu Duan, Ke Xue, Shengli An

**Affiliations:** 1 School of Rare Earth Industry, Inner Mongolia University of Science and Technology, Baotou 014010, China; 2022022101@stu.imust.edu.cn (J.Q.); xue_ke0@126.com (K.X.); 2 Key Laboratory of Green Extraction & Efficient Utilization of Light Rare-Earth Resources, Inner Mongolia University of Science and Technology, Ministry of Education, Baotou 014010, China; 15180741757@163.cn; 3 Inner Mongolia Key Laboratory of Advanced Ceramic Materials and Devices, Inner Mongolia University of Science and Technology, Baotou 014010, China; 4 Guizhou High-Level Institution Key Laboratory of High-Performance Battery Materials, Guizhou University, Guiyang 550025, China

**Keywords:** solid-state electrolyte, doping modification, Li_7_La_3_Zr_2_O_12_ (LLZO), ionic conductivity

## Abstract

Solid-state electrolytes (SSEs) have emerged as the most promising alternative to liquid electrolytes in batteries due to their enhanced stability and safety. Among these, the garnet-type Li_7_La_3_Zr_2_O_12_ (LLZO) solid electrolyte has attracted significant research interest due to its wide electrochemical stability window and good air stability. However, the ionic conductivity of LLZO is lower due to its high sintering temperature and unstable phase structure. In this study, Li_6.4+x_Fe_0.2_La_3_Zr_2−x_Bi_x_O_12_ (x = 0, 0.05, 0.1, 0.15) solid electrolytes were synthesized using a conventional solid-state reaction method by co-doping LLZO with Fe^3+^ and Bi^3+^ ions. Compared with pure LLZO, doping with Fe^3+^ effectively stabilizes the cubic phase, thereby enhancing the ionic conductivity. Moreover, Bi^3+^ doping significantly lowers the sintering temperature of the electrolyte, which in turn reduces energy consumption during the processing. The co-doping of Fe^3+^ and Bi^3+^ not only improves the density of the LLZO electrolyte, achieving a relative density of up to 95%, but also increases the ionic conductivity, with a maximum value of 7.57 × 10^−4^ S·cm^−1^ observed at the optimal composition (Li_6.4+x_Fe_0.2_La_3_Zr_2-x_Bi_x_O_12_, x = 0.1).

## 1. Introduction

All-solid-state batteries (ASSBs) offer superior stability and safety compared with conventional lithium-ion batteries (LIBs) with liquid electrolytes [1,2,3,4,5]. Among the various types of solid-state electrolytes, garnet-type electrolytes stand out due to their high lithium ionic conductivity and excellent battery performance. Among these, the Li_7_La_3_Zr_2_O_12_ (LLZO) garnet solid electrolyte has garnered significant attention due to its outstanding lithium-ion conductivity, wide electrochemical stability window, and enhanced safety [6,7,8,9,10]. LLZO has emerged as a promising candidate for replacing liquid electrolytes in solid-state batteries. Murugan et al. [11] first synthesized LLZO in 2007 and demonstrated that it exhibits a high lithium-ion conductivity of up to 3 × 10^−4^ S·cm^−1^ at room temperature, along with excellent thermal and chemical stability. As an ideal solid-state battery electrolyte, its electronic conductivity should remain at a low level. Extensive studies have demonstrated that the electronic conductivity of LLZO electrolytes is nearly five orders of magnitude lower than their ionic conductivity [12,13]. Compared with the predominant ionic conduction, the electronic contribution becomes essentially negligible. Since the initial synthesis of LLZO, extensive research has focused on improving its performance using various modification techniques [8,14,15]. LLZO garnet-type solid-state electrolytes possess both tetragonal (t-LLZO) and cubic (c-LLZO) phases, with the ionic conductivity of the cubic phase being one to two orders of magnitude higher than that of the tetragonal phase [16,17]. To stabilize the cubic phase and enhance its ionic conductivity, researchers have introduced dopants that substitute Li, La, or Zr sites in LLZO. Common dopants include Ga [18,19], Ge [17], Al [20,21], Y [22], Nb [23], and Cr [24], among others. These doping strategies are aimed at improving both the phase stability and ionic conductivity of LLZO, making it a more viable candidate for practical applications in ASSBs.

Despite significant progress in the development of LLZO solid-state electrolytes, several challenges remain. One of the key issues is the relatively low ionic conductivity of LLZO compared with liquid electrolytes, which limits its practical application in meeting daily energy demands. Additionally, LLZO exhibits poor sintering activity, requiring sintering temperatures above 1200 °C to achieve dense electrolytes. To address the low conductivity issue, researchers have focused on doping modifications. Huang et al. [25] successfully synthesized Li_7_La_3_Zr_1.9_Ta_0.1_O_12_ (LLZTO) by substituting Ta for Zr at the Zr site. Through the optimization of the Ta doping level, dense electrolyte ceramics were obtained, which not only stabilized the cubic phase but also improved ionic conductivity, achieving a value of 4.09 × 10^−4^ S·cm^−1^ at 25 °C. Similarly, Changbin Im et al. [26] synthesized Al-doped Li_6.875_La_3_Zr_2_O_12_ (Al-LLZO) by substituting Al for Li. Their study revealed that the Al doping stabilized the cubic phase of LLZO, shortened the Li(96h)-Li(24d) bond length, and reduced the activation energy for Li^+^ ion transport, thereby enhancing the ionic conductivity. Furthermore, Al doping mitigates the formation of Li_2_CO_3_ from the interaction of LLZO with air, thus improving the functionality of the grain boundaries.

Fe-doped LLZO (LFLZO) has emerged as one of the most promising dopants for enhancing ionic conductivity, particularly as a substitute for the Li site. Lu et al. [12] employed the density functional theory (DFT) to investigate the effects of Fe and Al dopants on LLZO. Their results demonstrated that Fe dopants retain more electrons, leading to an accumulation of positive charges on neighboring oxygen atoms. This reduces the binding energy of oxygen atoms to Li^+^ ions, thereby lowering the Li^+^ ion transport barrier and enhancing conductivity. In contrast, Al doping increases the electron density around the oxygen atoms, strengthening the oxygen–Li bond and thereby decreasing the ionic conductivity compared with Fe-doped LLZO.

Swantz et al. [27] found that the larger ionic radius of Bi^3+^ enhances the ionic conductivity of LLZO, while Bi doping significantly lowers the cubic-phase formation temperature of LLZO. Moreover, Bi substitution at the Zr site promotes explosive grain growth and alters the lattice parameter [28], further improving the ionic conductivity by facilitating Li^+^ ion migration. Co-doping with multiple elements has gained attention, as it tends to have a more pronounced effect on improving both the ionic conductivity and sintering activity compared with single-element doping. Zhou et al. co-doped Sr^2+^ and Mo^6+^ into LLZO to obtain the Li_6.6+x_La_3-x_Sr_x_Zr_1.8_Mo_0.2_O_12_ (x = 0–0.2) electrolyte. [29] Their findings indicated that Mo^6+^ doping stabilized the cubic phase, while Sr^2+^ doping improved both the ionic conductivity and relative density. The Li_6.65_La_2.95_Sr_0.05_Zr_1.8_Mo_0.2_O_12_ electrolyte exhibited an optimal ionic conductivity of 6.43 × 10^−4^ S·cm^−1^, with a 95% relative density at x = 0.05.

Wang et al. [30] successfully synthesized Ce^3+^ and Bi^3+^ co-doped LLZO by replacing La with Ce^3+^ and Zr with Bi^3+^ using the sol-gel method. The co-doped sample exhibited an improved microstructure, the lowest impedance, and the highest ionic conductivity of 5.12 × 10^−4^ S·cm^−1^, with a relative density of 93.9%. The electronic conductivity was found to be 10^−10^ S·cm^−1^, six orders of magnitude lower than the ionic conductivity. The activation energy was as low as 0.1083 eV, highlighting the fact that co-doping significantly enhances both the ionic conductivity and sintering activity of LLZO electrolytes. Moreover, co-doping efficiently promotes Li^+^ ion transport, making it a promising strategy for optimizing LLZO-based solid-state electrolytes. Kobi et al. [31] successfully synthesized Al and Mg co-doped garnet-type LLZO electrolytes via a sol-gel route, achieving an ionic conductivity of 4.7 × 10^−4^ S cm^−1^. This value represents a twofold enhancement compared with the LLZO samples doped solely with Al or Mg, highlighting the synergistic effects of dual-cation substitution on Li^+^ transport kinetics. However, the synthesis required a high sintering temperature of 1200 °C, which imposes significant energy consumption. Liu et al. [32] synthesized Ba, Y, and Al co-doped garnet-type LLZO electrolytes via a solid-state reaction method. Despite the optimization of the sintering temperature, the process still required an elevated temperature of 1190 °C, yielding a modest ionic conductivity of 2.96 × 10^−4^ S cm^−1^ and a relative density of 94.19%. These results suggest that the further refinement of sintering protocols may be necessary to improve the sintering activity of LLZO electrolytes and to mitigate grain boundary resistance and enhance densification in co-doped LLZO systems.

In the present study, Fe^3+^ and Bi^3+^ co-doped Li_6.4+x_Fe_0.2_La_3_Zr_2-x_Bi_x_O_12_ (x = 0, 0.05, 0.1, 0.15) solid electrolytes were synthesized using a conventional solid-state reaction method. In this approach, Bi^3+^ was substituted for Zr^4+^ at the Zr site, and Fe^3+^ was substituted for Li^+^ at the lithium site. The microstructure and ionic conductivity of the doped electrolytes were characterized and analyzed. The results indicated that the co-doping of Fe^3+^ and Bi^3+^ significantly enhanced the ionic conductivity of the LLZO-based electrolyte. The maximum ionic conductivity achieved was 7.57 × 10^−4^ S·cm^−1^ at the optimal doping composition of Li_6.5_Fe_0.2_La_3_Zr_1.9_Bi_0.1_O_12_ (LFLZBO). Moreover, the incorporation of Fe^3+^ and Bi^3+^ substantially lowered the temperature required for cubic phase formation in the LLZO garnet structure while also improving the sintering activity of the electrolyte.

## 2. Results and Discussion

### 2.1. Phase Analysis

**Figure 1:**
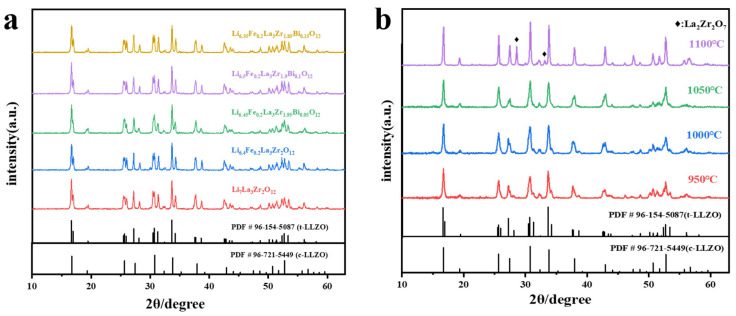
XRD patterns of (**a**) LLZO and Li_6.4+x_Fe_0.2_La_3_Zr_2-x_Bi_x_O_12_ (x = 0, 0.05, 0.1, 0.15) electrolyte powders calcined at 900 °C and (**b**) Li_6.5_Fe_0.2_La_3_Zr_1.9_Bi_0.1_O_12_ electrolyte pellets sintered at different temperatures.

The X-Ray diffraction (XRD) patterns of both the pure LLZO and Fe^3+^ and Bi^3+^ co-doped LLZO with varying electrolyte powder compositions after pre-treatment at 900 °C are shown in Figure 1a,. Following pre-sintering, the XRD patterns of the electrolyte powders reveal a predominant tetragonal phase, with no observable impurity phases, which is consistent with the standard diffraction pattern (PDF # 96-154-5087).

The XRD patterns of Li_6.5_Fe_0.2_La_3_Zr_1.9_Bi_0.1_O_12_ electrolyte pellets sintered at various temperatures are shown in Figure 1b. As the sintering temperature increases from 950 °C, distinct phase transitions are observed. At 950 °C and 1000 °C, some diffraction peaks exhibit splitting, indicating a partial transformation of the electrolyte’s structure from the tetragonal to the cubic phase due to the lower sintering temperature. Upon reaching 1050 °C, the XRD pattern of the electrolyte pellets reveals a fully developed cubic phase, with unimodal diffraction peaks and no detectable impurity phases. However, as the sintering temperature is further increased, impurity peaks appear in the XRD pattern, which is attributed to excessive Li volatilization at elevated temperatures. This results in the formation of the pyrochlore phase (La_2_Zr_2_O_7_) as a contaminant [33,34,35,36,37].

**Figure 2:**
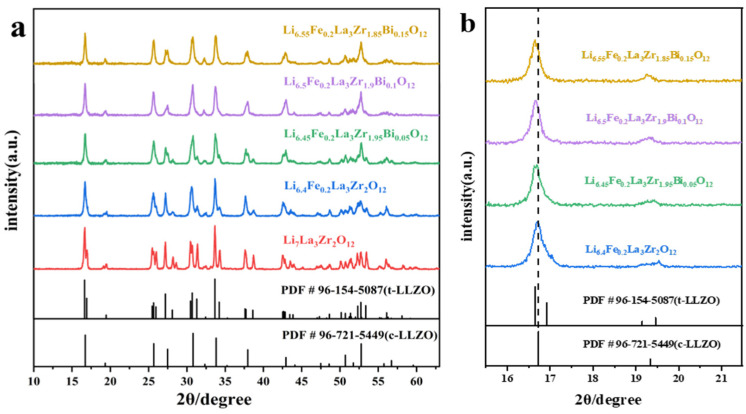
(**a**) XRD patterns of LLZO and Li_6.4+x_Fe_0.2_La_3_Zr_2-x_Bi_x_O_12_ (x = 0, 0.05, 0.1, 0.15) electrolyte pellets and (**b**) magnified XRD pattern of Li_6.4+x_Fe_0.2_La_3_Zr_2-x_Bi_x_O_12_ (x = 0, 0.05, 0.1, 0.15) electrolyte pellets.

The XRD patterns of Li_6.4+x_Fe_0.2_La_3_Zr_2-x_Bi_x_O_12_ (x = 0, 0.05, 0.1, 0.15) and LLZO electrolytes sintered at 1050 °C for 6 h are presented in Figure 2a. Fe^3+^ doping at the Li^+^ site results in a significant reduction in the intensity of the bimodal peaks in the XRD pattern, along with a pronounced shift from the tetragonal phase toward the cubic phase. These results suggest that Fe^3+^ doping effectively stabilizes the cubic phase of LLZO. Xiang et al. [38] also demonstrated the role of Fe^3+^ doping in stabilizing the cubic phase of LLZO. It is noteworthy that studies by Rettenwander et al. [39] have revealed that Fe, when incorporated into Li_7_La_3_Zr_2_O_12_ (LLZO), predominantly exists in the trivalent state (Fe^3+^), occupying identical crystallographic sites to Al^3+^ within the crystal lattice. This observation suggests that Fe^3+^ plays a role analogous to Al^3+^ in stabilizing the cubic phase of LLZO. Compositional analyses further demonstrate that Fe-doped garnet electrolytes maintain a chemically equilibrated state with a homogeneous elemental distribution of Fe. These findings collectively indicate that Fe incorporation does not compromise the electrochemical or structural integrity of LLZO electrolytes. Conversely, such doping effectively enhances the stabilization of the cubic phase structure in LLZO, thereby contributing to improved phase stability without inducing detrimental structural distortions [12].

The structure of the electrolyte progressively transitions to a cubic phase with increasing Bi^3+^ doping. A fully stabilized cubic phase is achieved when the Bi^3+^ doping concentration reaches 0.1. Further Bi^3+^ doping leads to the appearance of split peaks in the XRD pattern, indicating a shift from a unimodal to a bimodal peak profile. This suggests that the structure begins to transition from the cubic phase to a tetragonal phase. At a sintering temperature of 1050 °C, Li_6.5_Fe_0.2_La_3_Zr_1.9_Bi_0.1_O_12_ adopts a completely cubic phase. This is in contrast with both Li_7_La_3_Zr_2_O_12_ and Li_6.4_Fe_0.2_La_3_Zr_2_O_12_ [11,38,40,41,42], in which the sintering temperature must be significantly higher for the cubic phase to form, demonstrating the effective role of Bi^3+^ doping in lowering the required sintering temperature.

Figure 2b illustrates the magnified XRD patterns of Li_6.4+x_Fe_0.2_La_3_Zr_2-x_Bi_x_O_12_ (x = 0, 0.05, 0.1, 0.15). The XRD peaks shift to lower angles with increasing Bi^3+^ doping as compared with Li_6.4_Fe_0.2_La_3_Zr_2_O_12_. This shift is attributed to the larger ionic radius of Bi^3+^ (1.03 Å) relative to Zr^4+^ (0.84 Å), which leads to an expansion of the lattice parameter.

### 2.2. Analysis of the Valence of Fe and Bi

**Figure 3:**
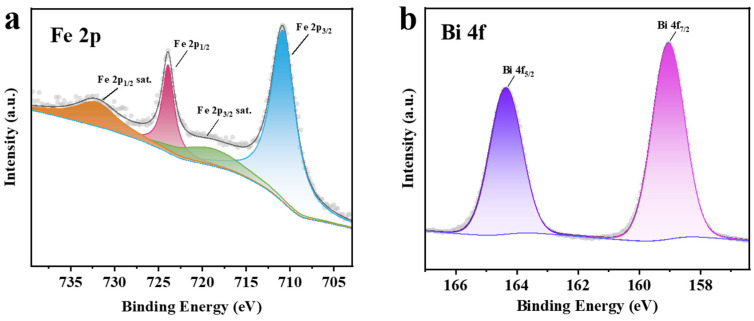
XPS spectra of the LFLZBO electrolyte: (**a**) Fe 2p and (**b**) Bi 4f regions.

To investigate the valence states of Fe and Bi in Li_6.5_Fe_0.2_La_3_Zr_1.9_Bi_0.1_O_12_ (LFLZBO), X-Ray photoelectron spectroscopy (XPS) was performed on the samples, as shown in Figure 3. The Fe 2p spectrum is presented in Figure 3a. As Fe is a transition metal, its XPS spectrum exhibits a characteristic spin-orbit splitting. The peaks observed at 710.8 eV and 724.0 eV correspond to Fe 2p_3/2_ and Fe 2p_1/2_, respectively, with satellite peaks appearing at 719.0 eV and 732.0 eV, which are indicative of the Fe^3+^ state. Existing studies [39] have further corroborated that Fe incorporated into the Li_7_La_3_Zr_2_O_12_ (LLZO) electrolyte predominantly stabilizes as Fe^3+^, occupying substitutional sites within the LLZO lattice. This valence state aligns conclusively with XPS spectroscopic analysis, where the binding energy signatures of Fe 2p_3/2_ and Fe 2p_1/2_ orbitals correspond unambiguously to the trivalent oxidation state. The absence of discernible satellite peaks or secondary phases in the XPS spectra confirms the redox stability of Fe^3+^ within the garnet structure under ambient and operational conditions. When Fe-doped LLZO is paired with an Fe-containing composite anode [43,44], the cross-interface synergistic interaction of the Fe elements enables the formation of chemical bonds between the Fe in LLZO and Fe-based active sites in the composite anode, thereby reducing interfacial impedance. Meanwhile, the shared Fe elements facilitate rapid lithium-ion transport across the electrolyte/electrode interface, significantly enhancing the performance of solid-state batteries.

Spin-orbit splitting, similar to Fe 2p, has been observed in the Bi 4f spectrum, as shown in Figure 3b. The binding energy peaks around 159.0 eV are assigned to Bi 4f_7/2_, while those around 164.3 eV correspond to Bi 4f_5/2_. These binding energies are consistent with the Bi^3+^ oxidation state.

The doping of Fe^3+^ in LLZO induces the formation of lithium vacancies, which stabilizes the cubic phase by disrupting the ordered arrangement of the lithium ions. This process also contributes to a more disordered ion arrangement within the crystal lattice. In contrast, the substitution of Zr^4+^ with Bi^3+^ enhances the lithium-ion concentration within the lattice, promoting continuity within the Li^+^ transport channels, thereby improving ionic conductivity. Additionally, the larger ionic radius of Bi^3+^ as compared with Zr^4+^ leads to an expansion of the lattice constant, which consequently increases the size of the Li^+^ migration channels, facilitating faster lithium-ion conduction and further enhancing the ionic conductivity of the electrolyte material.

### 2.3. Microscopic Morphology Analysis

**Figure 4:**
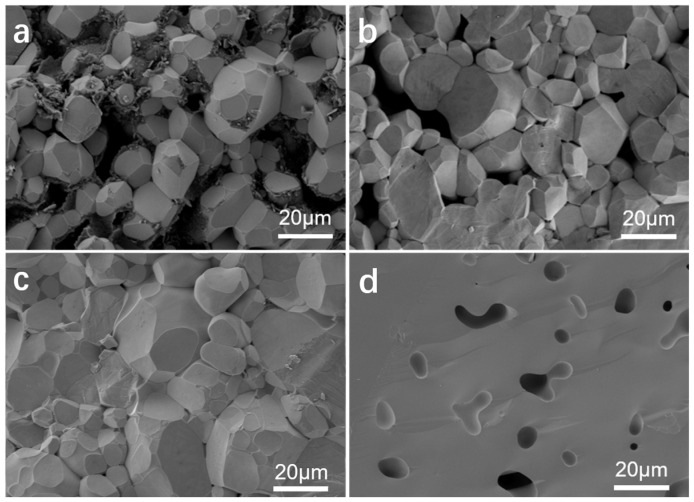
SEM images of the cross-sections of Li_6.5_Fe_0.2_La_3_Zr_1.9_Bi_0.1_O_12_ electrolyte pellets sintered at different temperatures: (**a**) 950 °C, (**b**) 1000 °C, (**c**) 1050 °C, and (**d**) 1100 °C.

The SEM images of the Li_6.5_Fe_0.2_La_3_Zr_1.9_Bi_0.1_O_12_ electrolyte pellets sintered at temperatures ranging from 950 °C to 1100 °C for 6 h are shown in Figure 4. At the lower sintering temperature of 950 °C, the grain structure is clearly visible in the cross-sectional image, exhibiting a rough surface, irregular size, and poor grain contact, with significant porosity at the grain boundaries. As the sintering temperature exceeds 1000 °C, the grain surfaces become smoother, the grain size increases slightly but remains non-uniform, and the grain boundary porosity decreases. At 1050 °C, the grains exhibit a more uniform polyhedral structure, with minimal porosity at the grain boundaries, improved grain contact, and the highest degree of ceramic densification. However, as the sintering temperature increases further, at 1100 °C, the grain boundaries completely fuse, and irregular, smooth voids form within the grains due to the Li volatilization at the elevated sintering temperature, resulting in the development of internal pores within the grains.

**Figure 5:**
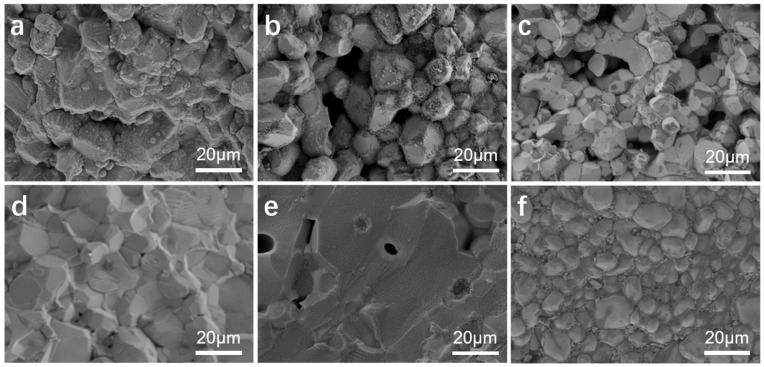
Cross-sectional SEM images of samples sintered at 1050 °C for 6 h: (**a**) pure LLZO, (**b**) Li_6.4_Fe_0.2_La_3_Zr_2_O_12_, (**c**) Li_6.45_Fe_0.2_La_3_Zr_1.95_Bi_0.05_O_12_, (**d**) Li_6.5_Fe_0.2_La_3_Zr_1.9_Bi_0.1_O_12_, (**e**) Li_6.55_Fe_0.2_La_3_Zr_1.85_Bi_0.15_O_12_, and (**f**) surface SEM image of Li_6.5_Fe_0.2_La_3_Zr_1.9_Bi_0.1_O_12_.

Figure 5 shows the SEM images of the cross-section of Li_6.4+x_Fe_0.2_La_3_Zr_2-x_Bi_x_O_12_ (x = 0, 0.05, 0.1, 0.15) sintered at 1050 °C for 6 h and the surface morphology of the Li_6.5_Fe_0.2_La_3_Zr_1.9_Bi_0.1_O_12_ ceramic pellets. In Figure 5a, the cross-sectional image of pure LLZO reveals numerous voids between the grains and a very rough grain surface, indicating a relatively low ceramic density of 83.4%. When Li^+^ is substituted with Fe^3+^, the grain surface becomes smoother, the grain size becomes more uniform, and the grain boundaries become more apparent, although substantial porosity remains between the grains (see Figure 5b). Upon doping with Bi^3+^, the grain boundaries start to shrink, and the surface roughness of the grains diminishes. At x = 0.1, the electrolyte pellet density reaches its maximum value of 95.1% (see Figure 5d). As the Bi^3+^ doping level increases further (x = 0.15), the grain boundaries are nearly absent, as seen in Figure 5e. However, the sample density decreases due to an increase in the voids between the grains and the formation of pores within the grains, which are likely caused by the Li volatilization at higher doping concentrations. Figure 5f shows the surface morphology of the Li_6.5_Fe_0.2_La_3_Zr_1.9_Bi_0.1_O_12_ electrolyte, which exhibits good densification, with uniform grain size and close grain contact.

**Figure 6:**
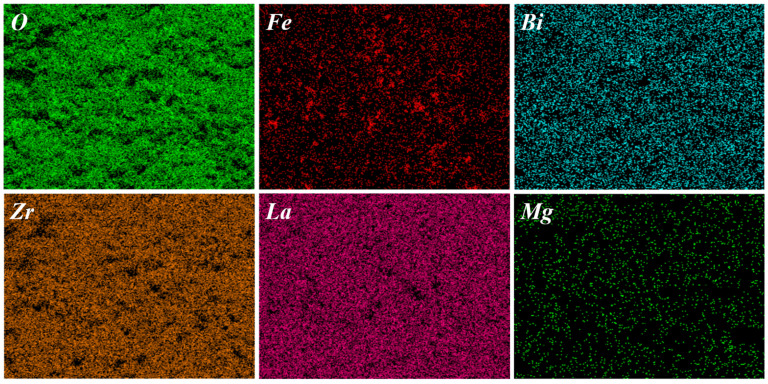
EDS spectrum of the Li_6.5_Fe_0.2_La_3_Zr_1.9_Bi_0.1_O_12_ sample.

Figure 6 displays the EDS elemental mapping images of the Li_6.5_Fe_0.2_La_3_Zr_1.9_Bi_0.1_O_12_ electrolyte sample. All constituent elements exhibit a homogeneous spatial distribution without detectable aggregation. As summarized in Table 1, the sample contains a trace amount of Mg (0.11%). Notably, Mg incorporation effectively inhibits the reaction of LLZO with atmospheric H_2_O and CO_2_, thereby enhancing its air stability [31].

### 2.4. Ion Conductivity Analysis

**Figure 7:**
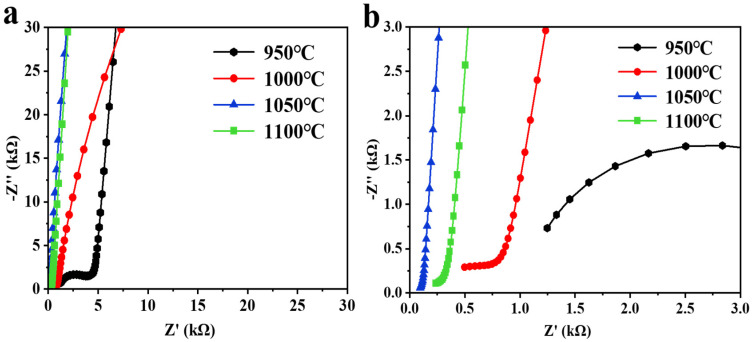
(**a**) Electrochemical impedance spectroscopy (EIS) spectra of the Li_6.5_Fe_0.2_La_3_Zr_1.9_Bi_0.1_O_12_ electrolyte at various sintering temperatures at a test temperature of 30 °C, and (**b**) magnified view of the EIS spectra of Li_6.5_Fe_0.2_La_3_Zr_1.9_Bi_0.1_O_12_.

The electrochemical impedance spectra of Li_6.5_Fe_0.2_La_3_Zr_1.9_Bi_0.1_O_12_ ceramic pellets at different sintering temperatures are shown in Figure 7. The Nyquist plots of LLZO typically exhibit two distinct semicircles in the high-frequency region, which correspond to the grain resistance and grain boundary resistance, and a sloping line in the low-frequency region, representing the blocking response of the silver electrode to the lithium ions [45]. This is a characteristic behavior of LLZO-based solid electrolytes: the semicircles corresponding to the grain and the grain boundary resistances are often not clearly discernible in the impedance spectra. This absence can be attributed to the narrow detection range (0.1 Hz–1 MHz) of the impedance spectroscopy instrument, which may cause some arcs to fall outside the detectable frequency range. As shown in Figure 7a, at 950 °C, a large semicircular arc appears in the electrochemical impedance spectroscopy (EIS) spectrum due to the low density of the ceramic pellets and the presence of a minor fraction of the tetragonal phase in the sample. At this temperature, the ionic conductivity is 3.90 × 10^−5^ S·cm^−1^.

As the sintering temperature increases, the ceramic density improves, and the tetragonal phase gradually transitions to the more stable cubic phase. As depicted in Figure 7b, the ionic conductivity reaches its maximum value of 7.57 × 10^−4^ S·cm^−1^ at 1050 °C, coinciding with the minimum impedance value. However, at higher sintering temperatures (1100 °C), lithium volatilization leads to the formation of voids in the ceramic grains, resulting in a decrease in ceramic density and a reduction in ionic conductivity. The corresponding EIS data indicate an increase in impedance at this temperature.

**Figure 8:**
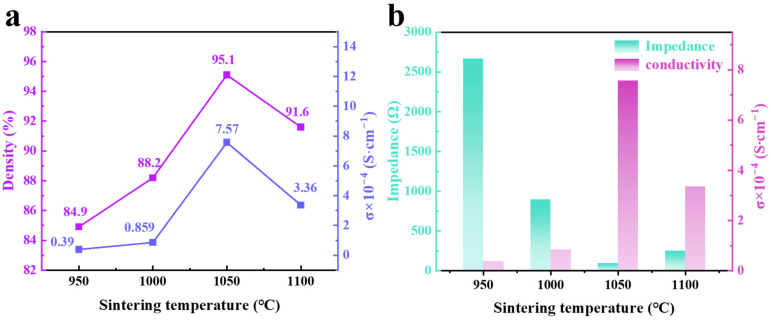
The effect of different sintering temperatures on the properties of Li_6.5_Fe_0.2_La_3_Zr_1.9_Bi_0.1_O_12_ pellets: (**a**) ionic conductivity (σ) and ceramic density, and (**b**) impedance and ionic conductivity (σ).

The density of the ceramic samples was determined using the Archimedes’ method, and their ionic conductivities were calculated by fitting the EIS spectra. These results are shown in Figure 8. Below 1050 °C, the ceramic density remains below 90% due to significant porosity at the grain boundaries and loose grain contacts. At 1050 °C, the porosity decreases, and the grains achieve closer contact, allowing the density to reach 95% (see Figure 8a). However, at 1100 °C, the presence of pores in the grains due to Li volatilization reduces the ceramic density, leading to an increase in electrolyte impedance and a decrease in ionic conductivity (see Figure 8b).

**Figure 9:**
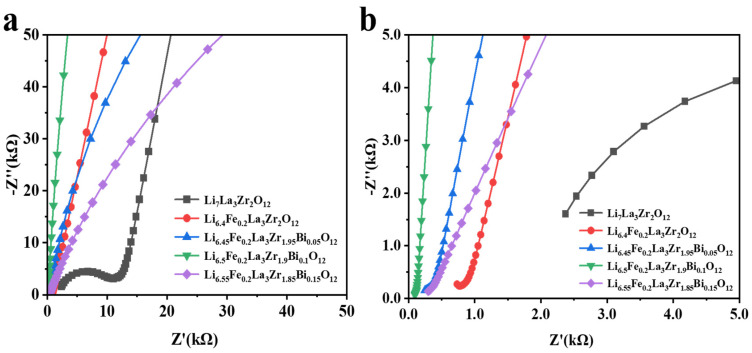
(**a**) Electrochemical impedance spectroscopy (EIS) spectra of LLZO and Li_6.4+x_Fe_0.2_La_3_Zr_2-x_Bi_x_O_12_ (x = 0, 0.05, 0.1, 0.15) electrolytes at a test temperature of 30 °C, and (**b**) magnified view of the EIS spectra of LLZO and Li_6.4+x_Fe_0.2_La_3_Zr_2-x_Bi_x_O_12_ (x = 0, 0.05, 0.1, 0.15) electrolytes.

Figure 9 presents the electrochemical impedance spectroscopy (EIS) data for both undoped LLZO and the Bi^3+^/Fe^3+^ co-doped Li_6.4+x_Fe_0.2_La_3_Zr_2-x_Bi_x_O_12_ (x = 0, 0.05, 0.1, 0.15) solid electrolytes. The undoped LLZO, which primarily adopts the tetragonal phase, exhibits an ionic conductivity of 1.01 × 10^−5^ S·cm^−1^. This value aligns with previously reported ionic conductivities for tetragonal-phase LLZO, confirming the consistency of the results [46]. As shown in Figure 9a, Fe^3+^ doping promotes the stabilization of the cubic phase of LLZO, while the tetragonal phase gradually diminishes. The cubic phase of LLZO, characterized by a higher concentration of lithium vacancies, facilitates enhanced Li^+^ ion conduction due to the expansion of the Li^+^ transport pathways [47,48,49,50]. As a result, the enhanced ionic conductivity of the ceramic samples compared with pure LLZO is primarily attributed to the increased Li vacancy concentration induced by Fe^3+^ doping in the LLZO matrix, while the improved relative density of the specimens synergistically contributes to the overall conductivity enhancement through microstructural optimization. By varying the Bi^3+^ doping levels in Li_6.4_Fe_0.2_La_3_Zr_2_O_12_, the ionic conductivity can be further improved. The highest ionic conductivity of 7.57 × 10^−4^ S·cm^−1^ is observed when the Bi^3+^ doping level is approximately 0.1 (see Figure 9b). This improvement is attributed to the substitution of Zr^4+^ by Bi^3+^, which introduces additional lithium ions into the lattice, ensuring charge conservation and enhancing the lithium-ion continuity within the transport channels. Additionally, the larger ionic radius of Bi^3+^ (1.03 Å) compared with Zr^4+^ (0.84 Å) results in an increased lattice constant, which further widens the Li^+^ migration channels.

**Figure 10:**
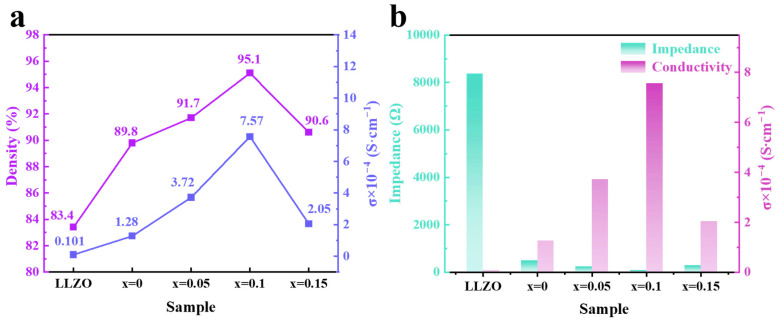
(**a**) Ionic conductivity (σ) and ceramic density and (**b**) impedance and ionic conductivity (σ) of LLZO and Li_6.4+x_Fe_0.2_La_3_Zr_2-x_Bi_x_O_12_ (x = 0, 0.05, 0.1, 0.15) pellets.

Figure 10 illustrates the relationship between density, ionic conductivity, and impedance for both LLZO and Li_6.4+x_Fe_0.2_La_3_Zr_2-x_Bi_x_O_12_ (x = 0, 0.05, 0.1, 0.15). As shown in Figure 10a, both the density and ionic conductivity of the electrolyte pellets increase with the doping concentration of Bi^3+^. However, when the Bi^3+^ doping level reaches 0.15, a significant amount of Bi^3+^ replaces Zr^4+^, leading to an increased lithium content in the lattice and a reduction in the concentration of lithium vacancies. This results in a decrease in ionic conductivity, which drops to 2.05 × 10^−4^ S·cm^−1^. Moreover, excessive Bi^3+^ doping lowers the densification temperature of the electrolyte pellets, causing over-sintering and significant lithium volatilization at elevated temperatures. This leads to the formation of pores within the electrolyte’s structure, reducing densification and increasing impedance (see Figure 10b).

**Figure 11:**
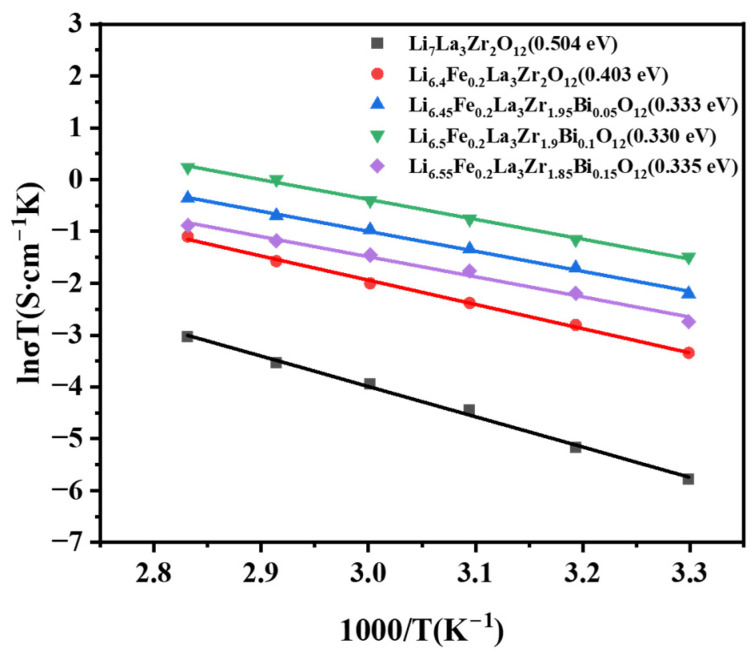
The Arrhenius spectrum of the ionic conductivity of the Li_6.4+x_Fe_0.2_La_3_Zr_2-x_Bi_x_O_12_ (x = 0, 0.05, 0.1, 0.15) solid electrolyte.

Figure 11 presents the Arrhenius plots of Li_6.4+x_Fe_0.2_La_3_Zr_2-x_Bi_x_O_12_ (x = 0, 0.05, 0.1, 0.15) garnet-type solid-state electrolytes. The undoped Li_7_La_3_Zr_2_O_12_ sample exhibits the highest activation energy (*E_a_*) of 0.504 eV, indicating that Li^+^ migration through the three-dimensional garnet lattice is significantly hindered by the elevated energy barrier, thereby resulting in reduced ionic conductivity. Upon Fe^3+^ substitution, the activation energy decreases markedly to 0.403 eV, suggesting enhanced Li^+^ mobility and diffusivity within the ionic transport channels. With increasing Bi^3+^ doping content, the activation energy initially decreases and then rises. At a Bi^3+^ doping level of x = 0.1 (Li_6.5_Fe_0.2_La_3_Zr_1.9_Bi_0.1_O_12_), the activation energy reaches a minimum value of 0.330 eV. This optimal doping concentration minimizes the energy barrier for Li^+^ migration in the cubic garnet structure, leading to the highest ionic conductivity. However, further increasing the Bi^3+^ content to x = 0.15 elevates the activation energy to 0.335 eV, signifying a decline in the Li^+^ mobility and diffusivity due to the excessive Bi doping, which causes the electrolyte to overburn and the porosity becomes higher, potentially causing lattice distortion or the blocking of conduction pathways.

## 3. Materials and Methods

### 3.1. Material Synthesis

Li_6.4+x_Fe_0.2_La_3_Zr_2-x_Bi_x_O_12_ (x = 0, 0.05, 0.1, 0.15) solid electrolyte powders were synthesized using a conventional solid-state reaction method. Initially, La_2_O_3_ (Aladdin, 99.99%, Riverside, CA, USA) was dried at 900 °C for 12 h. In accordance with the stoichiometric composition, LiOH·H_2_O (Aladdin, 99.0%, CA, USA), Fe_2_O_3_ (Aladdin, 99.9%, CA, USA), La_2_O_3_ (Aladdin, 99.99%, CA, USA), ZrO_2_ (Aladdin, 99.99%, CA, USA), and Bi_2_O_3_ (Aladdin, 99.9%, CA, USA) were then added to a ball mill. To compensate for lithium loss during sintering, an excess of 15% LiOH·H_2_O was included. The mixture was ball-milled for 24 h in isopropyl alcohol using agate balls as the grinding medium. After ball milling, the slurry was dried for more than 12 h at 80 °C in a blast-drying oven to remove all residual solvent.

For the pre-treatment step, the dried powders were placed into an MgO crucible and sintered at 900 °C for 6 h. The resulting powders exhibited a tetragonal phase, as confirmed by X-Ray diffraction (XRD). The pre-sintered powders were then ground in an agate mortar and sieved through a 200 mesh screen to obtain fine, uniform powder suitable for further processing.

Electrolyte pellets were fabricated by pressing the sifted powder into a mold with a 15 mm diameter. The master powder was placed at the bottom of an MgO crucible, and the electrolyte pellets were arranged on a platinum scaffold inside the crucible. To achieve compact ceramic pellets, the MgO crucible was covered with MgO round plates and sintered at temperatures ranging from 950 to 1100 °C for 6 h. After sintering, the electrolyte pellets were polished using sandpaper to achieve a smooth surface, with isopropyl alcohol used as the polishing solvent.

To prepare the symmetric cells for electrochemical measurements, silver (Ag) electrodes were deposited on both sides of the Li_6.4+x_Fe_0.2_La_3_Zr_2-x_Bi_x_O_12_ (x = 0, 0.05, 0.1, 0.15) electrolyte pellets, forming Ag/LFLZBO/Ag symmetric cells. The cells were then dried in an oven to remove any residual moisture before impedance measurements were conducted.

### 3.2. Characterization

The phases of the synthesized electrolyte powders and pellets were characterized using X-Ray diffraction (XRD) with a Rigaku MiniFlex-600 X-Ray diffractometer (Rigaku Corporation, Tokyo, Japan). The X-Ray source was Cu Kα radiation, operated at 40 kV and 40 mA, with a scanning rate of 2°/min over a 2θ range of 10° to 70°. The valence states of Fe and Bi in the Li_6.5_Fe_0.2_La_3_Zr_1.9_Bi_0.1_O_12_ (LFLZBO) electrolytes were analyzed using an ESCALAB 250Xi X-Ray photoelectron spectrometer (Thermo Fisher Scientific, Waltham, MA, USA).

The actual sintering density (*ρ*) of the ceramic electrolyte pellets was determined using the Archimedes method. Given that LLZO reacts with H_2_O to form an insulating phase [51,52], anhydrous ethanol was employed as the test medium instead of water. Ethanol, being chemically inert and exhibiting less reactivity with lithium ions in LLZO, demonstrates superior wettability due to its significantly lower surface tension compared with water. This property effectively reduces bubble adhesion at the solid–liquid interface and enhances the accuracy of volumetric measurements during Archimedes’ density characterization. The density was calculated using the following Equation (1):

(1) $$\;\rho =\frac{m_{0}}{m_{2}-m_{1}}\times \rho_{\omega }$$

where *m*_0_, *m*_1_, and *m*_2_ represent the dry weight, immersed weight, and saturated weight of the sample, respectively. The average value was obtained through multiple weighings, and $\rho_{\omega }$ represents the density of anhydrous ethanol (0.789 g/cm^3^). The relative density ($\rho_{\gamma }$) of the sample was calculated using the following Equation (2):

(2) $$\;\rho_{\gamma }=\frac{\rho }{\rho_{0}}\times 100\%$$

where $\rho_{0}$ is the theoretical density of the ceramic sample, which is 5.11 g/cm^3^ [53].

Electrochemical impedance spectroscopy (EIS) was employed to measure the impedance of the electrolyte samples. The measurements were conducted with an amplitude of 10 mV and a frequency range of 0.1 Hz to 1 MHz using an Ivium electrochemical workstation (Ivium Technologies, Eindhoven, Netherlands). The obtained data were fitted using Z-view 3.1 software. The ionic conductivity (σ) was calculated by substituting the impedance value (R), obtained from the Z-view 3.1 fitting, into the following Equation (3):

(3) $$\;\sigma =\frac{L}{R\times S}$$

where *L* represents the thickness of the electrolyte pellet and *S* is the area of the blocking electrode. After gold sputtering, the cross-sections of the electrolyte ceramics were characterized using scanning electron microscopy (SEM). The SEM employed was a field emission Carl Zeiss Sigma 300 (Carl Zeiss AG, Jena, Germany).

## 4. Conclusions

The tetragonal-phase garnet-type solid electrolyte Li_6.4+x_Fe_0.2_La_3_Zr_2-x_Bi_x_O_12_ (x = 0, 0.05, 0.1, 0.15) was successfully synthesized via the solid-state method at 900 °C. The powder was subsequently sintered at 1050 °C, a temperature significantly lower than the conventional sintering temperature for LLZO, resulting in dense electrolyte ceramic pellets. X-Ray diffraction (XRD) analysis confirmed that the sintered ceramics matched the cubic phase of the standard LLZO structure. The substitution of Fe^3+^ for Li^+^ stabilizes the cubic phase by introducing lithium vacancies within the crystal lattice, which facilitates Li^+^ migration. Furthermore, Bi^3+^ doping, which replaces Zr^4+^ due to its larger ionic radius, expands the Li^+^ transport channels and increases the number of transferable Li^+^ ions.

As a result, the ceramic pellets exhibited improved densification and a gradual increase in ionic conductivity. At room temperature, the sample with x = 0.1 demonstrated the highest ceramic density (95.1%) and ionic conductivity (7.57 × 10^−4^ S·cm^−1^), which corresponded to the maximum concentration of lithium vacancies and transferable Li^+^. This co-doping strategy of Fe^3+^ and Bi^3+^ significantly enhances the ionic conductivity of the LLZO solid electrolyte, offering substantial potential for advancing solid-state lithium-ion batteries. These findings contribute valuable insights into the optimization of solid-state electrolytes and provide a theoretical basis for further research and process improvements in next-generation energy storage technologies.

## Figures and Tables

**Table 1:** The Li_6.5_Fe_0.2_La_3_Zr_1.9_Bi_0.1_O_12_ sample’s elemental composition.

Element	Signal Type	Wt % Sigma	At %
O	EDS	0.13	70.15
Mg	EDS	0.04	0.11
Fe	EDS	0.12	1.12
Zr	EDS	0.19	10.81
La	EDS	0.24	17.26
Bi	EDS	0.19	0.55
Total content			100.00

## Data Availability

The data presented in this study are available on request from the corresponding author.
